# Reversion of pH-Induced Physiological Drug Resistance: A Novel Function of Copolymeric Nanoparticles

**DOI:** 10.1371/journal.pone.0024172

**Published:** 2011-09-26

**Authors:** Rutian Li, Li Xie, Zhenshu Zhu, Qin Liu, Yong Hu, Xiqun Jiang, Lixia Yu, Xiaoping Qian, Wanhua Guo, Yitao Ding, Baorui Liu

**Affiliations:** 1 The Comprehensive Cancer Center of Drum-Tower Hospital, Medical School of Nanjing University and Clinical Cancer Institute of Nanjing University, Nanjing, People's Republic of China; 2 Jiangsu Key Laboratory of Molecular Medicine, Medical School of Nanjing University, Nanjing, People's Republic of China; 3 Laboratory of Mesoscopic Chemistry and Department of Polymer Science and Engineering, College of Chemistry and Chemical Engineering, Nanjing University, Nanjing, People's Republic of China; 4 National Laboratory of Solid State Microstructure, Department of Material Science and Engineering, Nanjing University, Nanjing, People's Republic of China; 5 Department of Nuclear Medicine, Drum-Tower Hospital, Medical School of Nanjing University, Nanjing, People's Republic of China; RMIT University, Australia

## Abstract

**Aims:**

The extracellular pH of cancer cells is lower than the intracellular pH. Weakly basic anticancer drugs will be protonated extracellularly and display a decreased intracellular concentration. In this study, we show that copolymeric nanoparticles (NPs) are able to overcome this “pH-induced physiological drug resistance” (PIPDR) by delivering drugs to the cancer cells via endocytosis rather than passive diffussion.

**Materials and Methods:**

As a model nanoparticle, Tetradrine (Tet, Pka 7.80) was incorporated into mPEG-PCL. The effectiveness of free Tet and Tet-NPs were compared at different extracellular pHs (pH values 6.8 and 7.4, respectively) by MTT assay, morphological observation and apoptotic analysis in vitro and on a murine model by tumor volume measurement, PET-CT scanning and side effect evaluation in vivo.

**Results:**

The cytotoxicity of free Tet decreased prominently (***P***<0.05) when the extracellular pH decreased from 7.4 to 6.8. Meanwhile, the cytotoxicity of Tet-NPs was not significantly influenced by reduced pH. In vivo experiment also revealed that Tet-NPs reversed PIPDR more effectively than other existing methods and with much less side effects.

**Conclusion:**

The reversion of PIPDR is a new discovered mechanism of copolymeric NPs. This study emphasized the importance of cancer microenvironmental factors in anticancer drug resistance and revealed the superiority of nanoscale drug carrier from a different aspect.

## Introduction

Copolymeric nanoparticles (NPs) have been proved to be effective carriers for the delivery of antitumor agents with some of them had been brought into clinical use [1]–[3]. The superiority of most NPs without active targeting strategies was mainly attributed to the enhanced permeability and retention (EPR) effect [4]–[6] and the sustained release of drugs from the NPs [7]. In this report, by a series of in vitro and in vivo studies, we are trying to figure out another important mechanism of the copolymeric NPs: the reversion of pH-induced physiological drug resistance (PIPDR).

In addition to the genetic or epigenetic changes of cancer cells, the tumor microenvironment is also a critical factor that is attracting more and more attention in the latest years. That is because solid tumors are organ-like structures that are heterogeneous and structurally complex [8]. The altered physicochemical factors in the tumor microenvironment play a vital role in the resistance to anticancer drugs. The pH value of tumor tissue is a typical case in point. The extracellular pH value of cancer cells is lower than that in normal tissue whereas the intracellular pH of tumor is similar to that of normal tissue [9]. This unusual intracellular-extracellular pH gradient can influence the effect of weakly basic agents with an acid dissociation constant (Pka) of 7.5–9.5. These drugs will be protonated at the extracellular pH of tumors [8]. As the protonated forms of these agents are less membrane permeable, they will accumulate mostly outside the cell. The intracellular part of weakly basic drugs, under this condition, will not be able to reach a concentration high enough to kill cancer cells. Raghunand et al. defined this phenomenon as “physiological drug resistance”, as distinct from the “biochemical drug resistance” which is caused by the altered signaling pathway or expression of proteins. [10]. However, pH is not the only physiochemical factor that influences drug distributions in the tumor tissue; other factors such as penetration [11] or interstitial fluid pressure [8] also play such role. Therefore, in this paper we apply a more specified label to this phenomenon: “pH-induced physiological drug resistance (PIPDR).”

PIPDR plays an important role in chemotherapy failure first because this phenomenon occurs in most tumors [12], varying little with their genetic characteristics; second, an indispensable part of present chemotherapeutics are weakly basic drugs such as doxorubicin, mitoxantrone, vincristine and vinblastine, as well as paclitaxel and docetaxel [8], [13]. Studies have revealed that the cytotoxicity of these drugs is hampered at lower extracellular tumor pH [8], [10], [13]–[16].

A few attempts have been made to overcome PIPDR. CM Lee et al. [17] treated cancer cells with the co-administration of doxorubicin and chloroquine (a competing base) or omeprazole (H^+^ pump inhibitor). Raghunand et al. [15] enhanced the in vivo effectiveness of doxorubicin by the increase of tumor pH (adding sodium bicarbonate in the drinking water of mice). However, these methods rely on the use of additional agents (sometimes toxic), or changing the homeostasis of the body, which may cause inevitable side effects.

**Figure 1:**
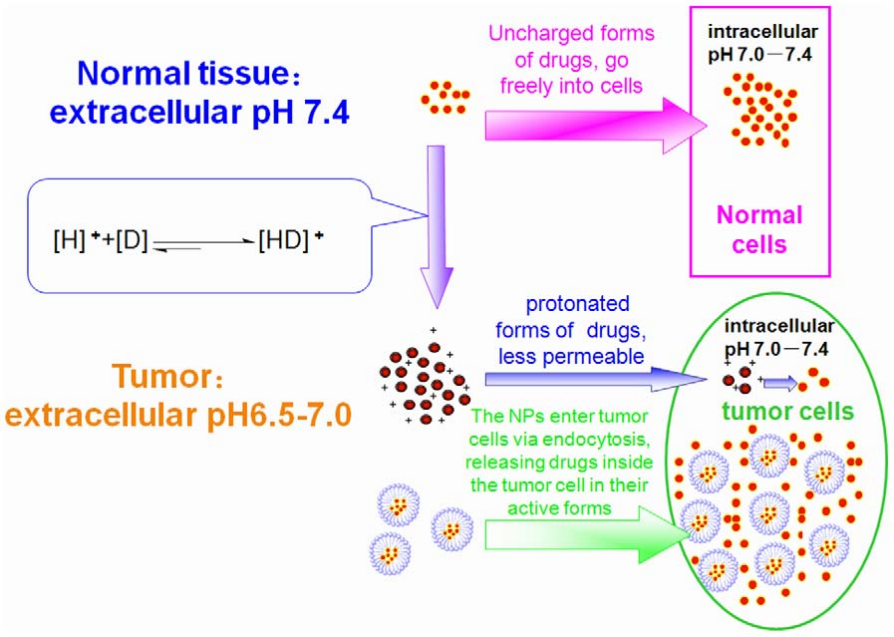
The cause of PIPDR and the reversion of PIPDR by NPs. [D] stands for drugs and [H]^+^ stands for the proton.

In this paper, we propose that polymeric NPs composed of amphiphilic block copolymers is a better candidate used to overcome PIPDR as it is more effective with less toxicity than other approaches. It is widely accepted that NPs enter cells via endocytic uptake and release the loading drugs intracellularly [18], [19]. As a result, when the weakly basic drugs are incorporated into the NPs, the drug inside will enter the cancer cell by endocytosis. The pH value inside the cancer cells was normal (around 7.4), basic drugs released in the cytoplasm would remain its active forms (uncharged forms) and kill the cancer cell effectively (Fig. 1.).

To verify our hypothesis, Tetrandrine (Tet), a bis-benzylisoquinoline alkaloid (Pka 7.80 and logP 5.78, a typical weakly base) isolated from the dried root of Hang-Fang-Chi (Stephania tetrandra S. Moore) [20] was incorporated into the copolymer made of methoxy poly (ethylene glycol)–polycaprolactone (mPEG-PCL) as a model. The antitumor effect of free Tet and Tet-loaded NPs (Tet-NPs) was compared at different extracellular pH values (pH 7.4 and pH 6.8, respectively). We also evaluated the antitumor efficacy of the NPs in tumor xenografts in a murine model with acidic and alkalinized extracellular pH to estimate the NPs' potential to reverse in vivo PIPDR. Both in vitro and in vivo studies revealed that the incorporation of Tet into mPEG-PCL nanoparticles effectively reversed PIPDR of tumors thus enhance the antitumor efficacy of Tet.

## Materials and Methods

### Materials

Tetrandrine (molecular formula C_38_H_42_N_2_O_6_, Molecular Weight 638.76) was obtained as a powder with a purity of >98% from Jiangxi Yibo Pharmaceutical Development Company ( Jiangxi, China). Methoxy-polyethyleneglycol (mPEG, MW: 4 kDa) were purchased Nanjing Well Chemical Co.Ltd. (P.R.China) and was dehydrated by azeotropic distillation with toluene, and then vacuum dried at 50°C for 12 h before use. ε- caprolactone (ε-CL, Aldrich, USA) was purified by drying over CaH_2_ at room temperature and distillation under reduced pressure. Coumarin-6 (Aldrich,USA), Stannous octoate (Sigma, USA), PVA (Polymerization Degree 500 and Alcoholization Degree 88%, Shanghai Dongcang International Trading Co. Ltd. Shanghai China), RPMI 1640 (Gibco, USA), calf blood serum, (Minhai Bioengineering, Lanzhou, China), and dimethylthiazoly-2,5-diphenyltetrazolium bromide (MTT, Amersco, USA) were used as received. Acetonitrile and Methanol (Merck, Germany) were of chromatogram grade. All the other chemicals were of analytical grade and were used without further purification.

### Synthesis and characterization of mPEG-PCL copolymer and NPs

The mPEG-PCL block copolymer was synthesized from Methoxy-polyethyleneglycol (mPEG), (M_W_: 4 kDa) and ε- caprolactone (ε-CL) by a ring opening copolymerization and was characterized by ^1^HNMR, Gel permeation chromatography according to our previous study [20]. The Tet-loaded NPs (Tet-NPs) and blank NPs were prepared from the mPEG-PCL copolymer by single O/W emulsion and solvent evaporation method also according to our previous study [20]. The particle size, morphology, stability, drug loading content and encapsulation efficiency of the NPs were evaluated (details in Methods S1).

To determine a drug release profile of Tet from the NPs at different pH values, the lyophilized Tet-NPs were suspended in 1 mL 0.01 M phosphate buffered saline (PBS) at pH 7.4 and 6.8 respectively to form the solutions containing Tet 500 µg/ml (loaded in NPs, thus the solubility was substantially improved). The solution was then placed into a pre-swelled dialysis bag with a 12-kDa molecular weight cutoff (Sigma) and immersed into 10 ml 0.01 M PBS of pH 7.4 and pH 6.8 at 37°C. The PBS out of the dialysis bag were taken out and replaced by 10 mL of fresh PBS at the predetermined time interval. The concentrations of Tet were determined using HPLC (^18^C column, Agilent Technologies, Ltd., the mobile phase consisted of methanol: water = 9∶1, with 0.5% Triethylamine. Flow rate of 1 mL/min, the column temperature was 25°C, the injection volume was 20 µL, the detector was fixed at a wavelength of 280.0 nm.)

### *In vitro* studies

The in vitro cytotoxicity of free Tet, Tet-NPs at different extracellular pH values (pH 6.8 and pH 7.4) was determined by standard MTT assays in different cell lines. Human gastric carcinoma cell line BGC-823 and MKN-28, human colon cancer cell line LoVo, human hepatocellular carcinoma cell line HepG_2_ and mouse liver cancer cell line H_22_ were obtained from Shanghai Institute of Cell Biology (Shanghai, China). Human Umbilical Vein Endothelial Cells (HUVEC) were isolated from umbilical veins and cultured by our lab.

The tumor cells were grown routinely in a humidified atmosphere with 5% CO_2_. The culture medium was RPMI 1640 medium (pH 7.4) supplemented with 10% calf blood serum and changed every other day. The culture medium of HUVEC was EGM supplemented with 10% fetal bovine serum. The toxicity of the blank NPs was also determined by MTT assays at different extracellular pHs (The culture medium was adjusted to pH 7.4 and pH 6.8 with 1 M HCL or 1 M NaOH.)

The apoptosis assay was performed using an Annexin V-FITC Apoptosis Detection Kit (BioVision, Mountain View, CA) according to the manufacture's instructions. Briefly, cells were cultured in 60-mm Petri disks under pH 7.4 and pH 6.8 respectively and allowed to grow to 75–80% confluency. They were then exposed to free Tet and Tet-NPs at the concentration of 32.0 µM and compared with control cells treated with RPMI1640 or blank NPs for 24 hrs. Then cells were collected and incubated with Annexin-V-FITC for determining surface exposure of phosphatidyl serine in apoptotic cells. Analyses were performed with a FACScan Flow cytometer (Becton Dickinson, Sunnyvale, CA, USA).

### Particle cellular uptake studies

For the cell uptake experiment, LoVo and MKN-28 cells were seeded onto the glass covers placed in a 6-well plate with RPMI 1640 supplemented with 10% calf blood serum at the density of 5×10**^5^** cells/well. After incubation at 37°C in a humidified atmosphere with 5% CO_2_ for 24 hrs, cells were exposed to medium containing loaded NPs (equaled to coumarin-6 12.5 µg/mL in medium. When loaded in NPs, the solubility of coumarin-6 was substantially improved). After incubation for 4 hrs, the suspension was removed and cover glasses were washed 3–4 times with PBS at 4°C and 37°C sequentially. Then the cells were observed under a fluorescence microscopy (Zeiss imager A1, excitation 494 nm, emission 518 nm, magnification = 200×).

### *In vivo* antitumor efficacy evaluation

The animal experiments were performed following the guidelines in the Guide for the Care and Use of Laboratory Animals published by the US National Institutes of Health (NIH publication No. 85-23, revised 1985) and was approved by the Ethics Review Board for Animal Studies of Drum Tower Hospital, Medical School of Nanjing University (DTH ERMA 66.01/210A/2008).

**Figure 2:**
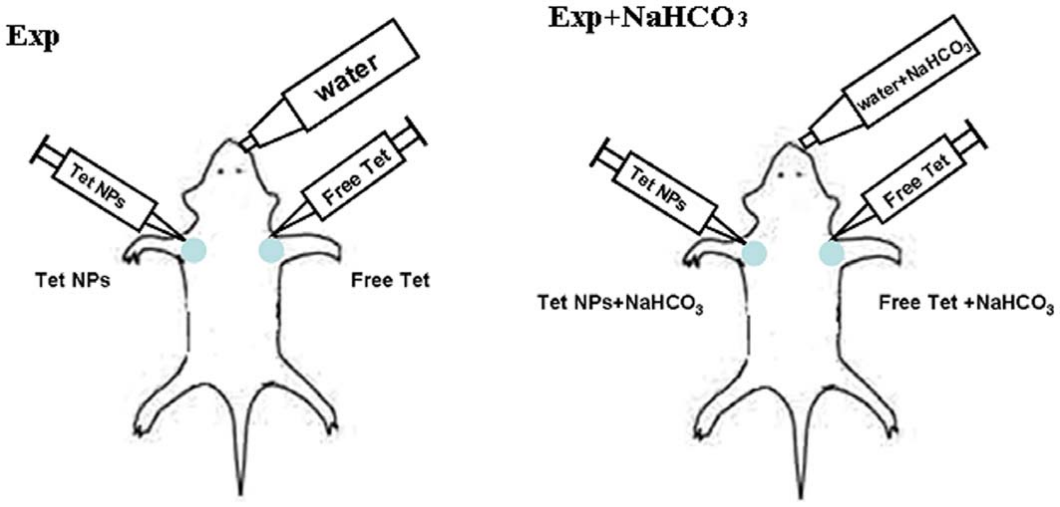
Administration protocol for in vivo study. The mice were injected s.c. at both of the axillary spaces with 4–6×10^6^ H_22_ cells. When the tumors were 50–150 mm^3^ in volume, the mice were randomly divided into two groups. One group of the mice were given 200 mM NaHCO_3_-supplemented water to drink ad libitum since then. The mice were treated with free Tet and Tet-NPs respectively at both axillary spaces intratumorally (for details, see Table 1.).

Male ICR mice were injected subcutaneously at both of the axillary spaces with 4–6×10^6^ H_22_ cells in saline. When the tumors reached 100–300 mm^3^ in volume, the mice were randomly divided into four groups, with each group being composed of 6 mice. Two groups of the mice were given 200 mM NaHCO_3_-supplemented water to drink ad libitum since then. The mice were treated with different Tet formulations at both axillary spaces intratumorally according to Table 1. and Fig. 2. The day of Tet administration was marked as Day0. Animal weights and tumor measurements were recorded every other day. The tumor volume was calculated by the formula (W^2^×L)/2, where W is the tumor measurement at the widest point, and L is the tumor dimension at the longest point.

**Table 1:** Administraion Protocol.

Group	Bicarbonate	Left axillary spaces	Right axillary spaces
Con	-	Saline (Con)	Blank NPs (Blank Nps)
Exp	-	Free Tet 15 mg/kg (Free Tet)	Tet-NPs 15 mg/kg (Tet-Nps)
Con+NaHCO_3_	200 mM NaHCO_3_	Saline (Con NaHCO_3_)	Blank NPs (Blank Nps+NaHCO_3_)
Exp+NaHCO_3_	200 mM NaHCO_3_	Free Tet 15 mg/kg (Free Tet+NaHCO_3_)	Tet-NPs15 mg/kg (Tet-Nps+NaHCO_3_)

There are totally four groups and eight subgroups in this study. Each group can be divided to two subgroups according to the agents injected to tumors in different axillary space. The name of each subgroup was indicated in the brackets. (Also see Fig. 2.)

For PET-CT imaging, one mouse from each group was taken randomly and fasting for 8 hrs before the imaging on Day 5. The mice were kept in the anesthetized condition during the whole PET-CT scanning procedure. Around 600 uCi of ^18^F-FDG was injected via the tail vein as a radiotracer for imaging. The imaging studies were performed with a combined PET-CT scanner for clinical use (Jemini JXL, Philips, USA). High resolution PET images were acquired with the mice 15–30 min after the administration of ^18^F-FDG, and the same field of view was covered as for CT. PET images were corrected for attenuation and scatter on the basis of the CT data. The image fusion was performed by an automatic image fusion system, using vendor-supplied software.

The Regions of Interest (ROI) was defined by three radiologists independently and maximum FDG uptake values in the tumor were obtained for the standard uptake value (SUV) calculated according to the following formula:
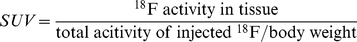



### Statistical analysis

Statistical analyses of data were done using Student's t test. The data are listed as Mean±SD, and values of ***P***<0.05 were accepted as a statistically significant difference.

## Results

### Copolymer synthesis and characterization of the copolymers and NPs

The number-average molecular weight (M_n_) of the copolymer determined by ^1^H-NMR was 23691(Fig.S1.and Table.S1.). The M_n_ and weight-average molecular weight (M_w_) of the copolymer determined by GPC were 25826 and 43763, respectively (Fig.S2. and Table.S1.). The polydispersity of the copolymer was 1.69 (Table.S1.). (Details in Results S1).

The diameter of the lyophilized Tet- NPs was 285.8 nm with a polydispersity around 0.054 (Table.S2.). The NPs exhibited a regular spherical shape and the size was around 300 nm when observed by TEM and AFM (Fig.S3.). The maximum drug loading content was 14.38% (Table.S3.).(Details in Results S1).

### *In vitro* release of Tet-NPs at different pH values

**Figure 3:**
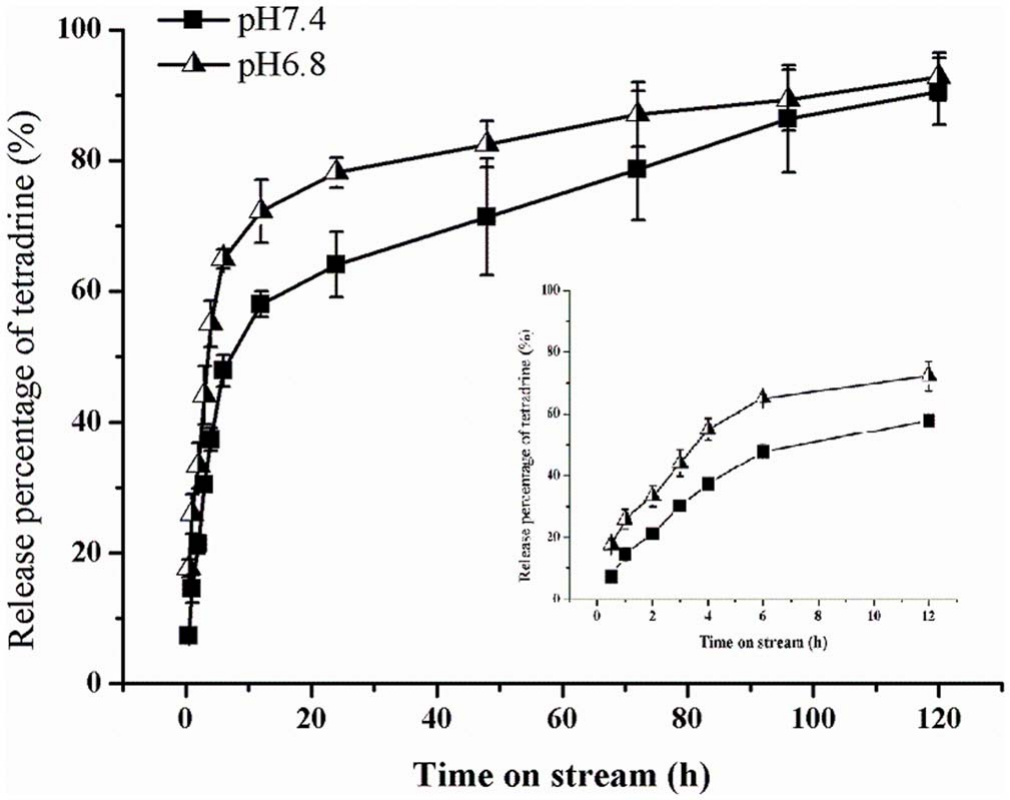
In vitro release profile of Tet from the NPs at different pH values. The data were presented as Mean±SD. Where bars are not shown, SD is less than the height of the points. The figure at the right shows the release profile at the first 12 h as it is not very clear at the original figure.

Fig. 3. shows the cumulative in vitro release profiles of the NPs at 37°C in PBS (pH 7.4 and pH 6.8, respectively). At the first 4 hrs, the NPs at both pH values exhibited a burst release. There was a burst release of more than 30% Tet at pH 7.4 and more than 50% Tet at pH 6.8. Then Tet in the NPs in both groups showed a sustained release manner with the release of Tet at pH 7.4 more sustainable. At 24 h, the release proportions at pH 7.4 and pH 6.8 were 78% and 64% respectively and at 120 h, more than 90% Tet was released in both groups.

### Copolymeric NPs can reverse PIPDR in cancer cell lines in vitro

**Figure 4:**
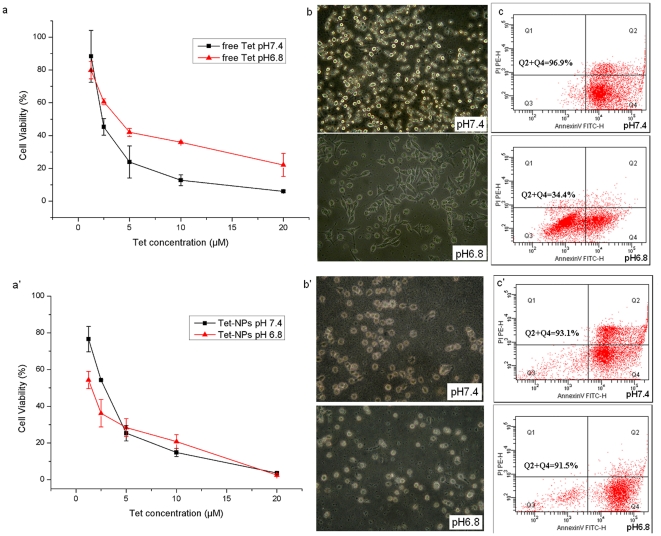
The PIPDR of MKN-28 cells to Tet (**Fig. 4.a–c**) and the reversion of PIPDR to Tet by incorporating it into PEG-PCL NPs (**Fig. 4.a′–c′**). The cytotoxicity of free Tet at different extracelluar pH values. pH 7.4 is the normal pH value used in cell culture while pH 6.8 is the pH value in most tumor tissues. Similar results can be found in other 3 different digestive cancer cells (Table.S4.). a. The morphological changes of MKN-28 cells at different extracelluar pH values. (400×). More cells remained normal morphology at lower extracellular pH. b. The apoptosis assay of MKN-28 cells treated with Tet at different extracelluar pH values. When the extracellular pH value decreased from 7.4 to 6.8, a prominent decrease in apoptotic ratio could be observed. a′ The cytotoxicity of Tet-NPs at different extracelluar pH values. pH 7.4 is the normal pH value used in cell culture while pH 6.8 is the pH value in most tumor tissues. Similar results can be found in other three different digestive cancer cells (Table.S4.). b′ The morphological changes of MKN-28 cells at different extracelluar pH values. (400×). The morphologic changes of MKN-28 cells were similar at both pH values. c′ The apoptosis assay of MKN-28 cells treated with Tet-NPs at different extracelluar pH values. The apoptotic ratios of the cells didn't change with the decrease of extracellular pH.

Fig. 4.a–c. shows the PIPDR of free Tet on MNK-28 cell line. It can be noticed from Fig. 4.a. that when the extracellular pH decreased from 7.4 to 6.8, the cytotoxicity of free Tet was hampered significantly (which was also evaluated by increase of IC_50_ values, ***P***<0.05, details in Table 2.). The morphologic changes of MKN-28 cells treated with free Tet at different pH values (Fig. 4.b) also shows that after treated with Tet for 24 hrs, most of cells at pH 7.4 shrank and turned round. However, at pH 6.8, an indispensable part of cells remain their normal shapes. The difference of the morphologic changes was confirmed by the apoptosis assay. Fig. 4.c showed that at pH 7.4, 96.9% of the MKN-28 cells were apoptotic whereas only 34.4% cells get apoptotic at a lower extracellular pH.

**Table 2:** *In vitro* cytoxicity of Tet-loaded nanoparticles and free Tet.

Cell lines	Formulation	IC50 (µM)	
		pH 7.4	pH 6.8
BGC-823	Free Tet	7.84±2.41	13.24±1.70*
	Tet-NPs	7.09±0.43	8.15±0.88#
MKN-28	Free Tet	7.66±2.07	11.71±0.87*
	Tet-NPs	6.57±0.60	3.90±1.42* #
Lovo	Free Tet	4.67±0.76	6.81±0.89*
	Tet-NPs	12.35±2.64#	3.05±0.53* #
HepG2	Free Tet	8.64±1.95	12.51±1.97*
	Tet-NPs	3.76±0.17#	4.12±0.50#
H_22_	Free Tet	8.82±0.80	14.68±3.39*
	Tet-NPs	15.69±0.43#	7.80±1.41* #

Data represent mean value±SD, n = 3.

*P<0.05, pH 6.8 vs pH 7.4 for the same formulation (Free Tet or Tet-NPs respectively).

# P<0.05,Tet-NPs vs Free Tet for the same pH value (pH 6.8 or pH 7.4).

It can be noticed from the table that when the extracellular pH decreased from 7.4 to 6.8, the cytotoxicity of free Tet in all the tumor cell lines attenuated (which was evaluated by increase of IC50 values, P<0.05). On contrary, the IC50 of Tet-NPs in none of the cell lines increased significantly.

Furthermore, at pH 6.8, the cytotoxicity of Tet-NPs in all the cell lines was more prominent than free Tet (P<0.05).

When the same experimental process was conducted using Tet-NPs, the results were quite different. According to Fig. 4.a′, when MKN-28 cells were treat with Tet-NPs, the cytotoxicity of Tet-NPs was not significantly influenced by the change of extracellular pH value (for IC_50_s at different pH values, ***P***>0.05, See Table 2.). The morphologic changes of the cells as well as the apoptotic proportion of cells treated with Tet-NPs under different pH values were also similar (Fig. 4.b′, Fig. 4.c′).

Similar results were also obtained on other 4 cancer cell lines. (Table 2.)

### Tet-NPs can reverse PIPDR and attenuated toxicity of Tet *in vivo*

**Figure 5:**
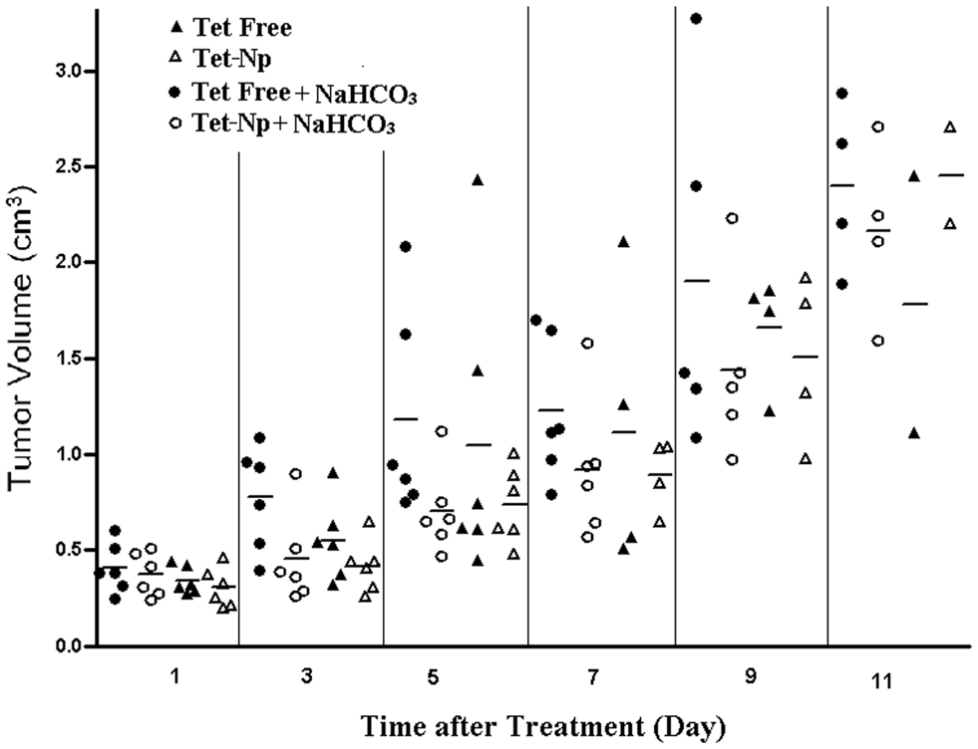
Tumor volumes comparison in the experimental groups. The tumor volumes were measured every other day. Each symbol represents an individual mouse; small horizontal lines indicate the mean.

Fig. 5. and Fig.S4. shows the growth of tumors in the experiment groups. At dosage of 15 mg/kg, both free Tet and Tet-NPs could delay the growth of tumor in a comparatively mild manner (with the highest tumor inhibition rate around 50%). In the subgroups treated with or without NaHCO_3_, Tet-NPs exhibited superior antitumor effect over free Tet. When the antitumor effect of the same formulation at different pH values was compared, free Tet exhibited better antitumor effect in the mice without NaHCO_3_ over those with NaHCO_3_. For the mice treated with Tet-NPs, there wasn't prominent difference caused by NaHCO_3_ supplement.

**Figure 6:**
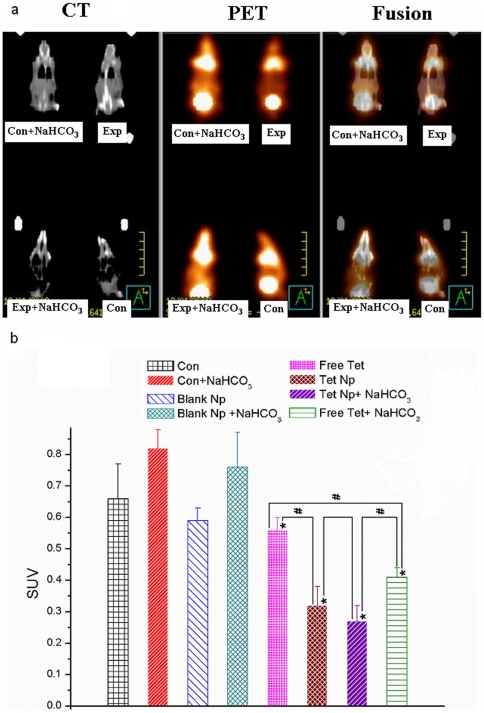
PET-CT scanning of the mice at the fifth day after treatment. a. For each mouse, the tumor at the left side was injected with saline or free Tet while the tumor at the right was administrated with Blank or Tet-NPs. b. The SUV values of each group. * indicates that ***P***<0.05 between the Exp/Blank groups and Con groups. (Exp/Blank vs Con, Exp+NaHC_O3_/Blank NaHC_O3_ vs Con NaHC_O3_). # indicates that ***P***<0.05 between each two groups linked by lines.

The antitumor effect was also evaluated by PET-CT to assess the metabolic changes in the tumors at the fifth day after drug administration (Fig. 6.). The metabolic activities of the tumor were measured semi-quantitatively by SUVs. As to Fig. 6., The two Tet formulations at both pH values reduced the metabolism of the tumor significantly (***P***<0.05). At both pH values, the tumors treated with Tet-NPs reduced the SUVs more drastically than free Tet (***P***<0.05). For the same formulation, when pH increased, free Tet exhibited better antitumor effect (SUV _Free Tet_>SUV _Free Tet+NaHCO3_ ***P***<0.05). However, the SUVs in tumors treated with Tet NP didn't change significantly (***P***>0.05).

**Figure 7:**
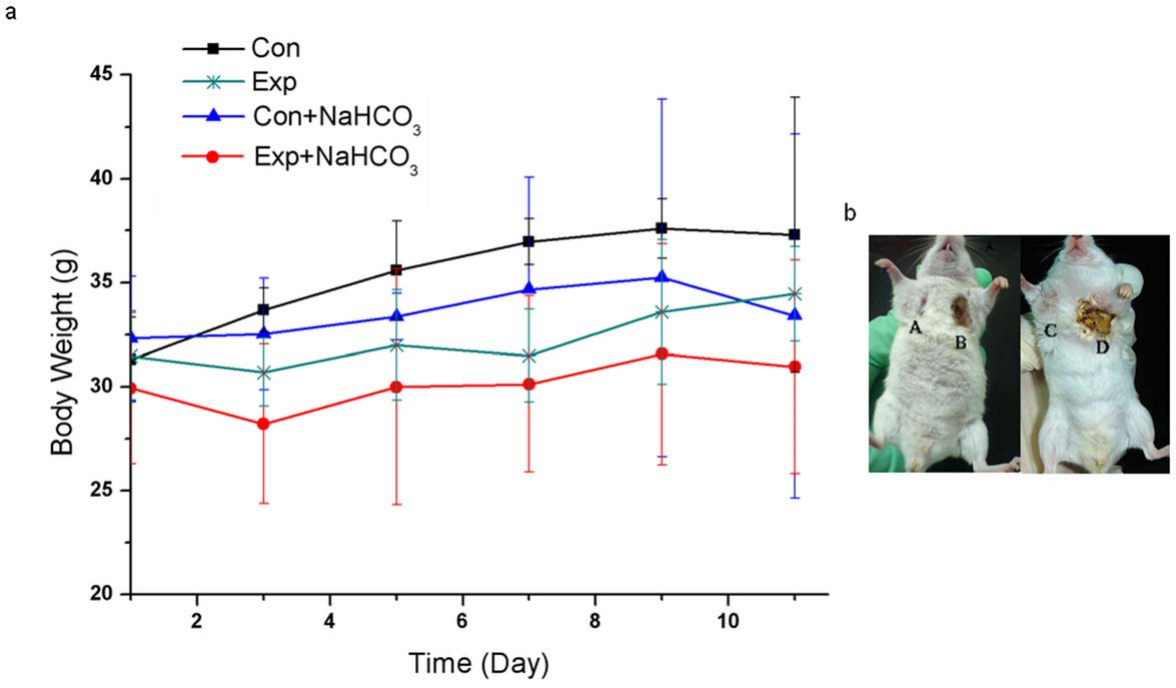
The side effects of Free Tet and Tet-NPs. a. The body weight change of the mice during therapy under different treatments. Body weight loss in this study is not an ideal parameter for the evaluation of wide effects in this study because every mouse received two different treatments. Nevertheless, the body weight of the Exp+NaHCO_3_ group was much lower than in the Con+NaHCO_3_ group while the differences of body weight between the Exp and Con group was not so obvious. b. Photos of the ulceration taken at the 7th day after administration. (A. Tet-NPs pH 6.8, B. Free Tet pH 6.8, C. Tet-NPs pH 7.4 and D. Free-Tet pH 7.4). Ulcerations were evaluated in details in Fig.S5.

Both local and systemic side effects were found in this study. The tumors treated with free Tet suffered from severe skin ulceration while Tet-NPs didn't cause obvious ulcerations (Fig. 7.b. The severity of the ulceration was scored according to its area, see Table.S4.). Free Tet also caused systemic toxicity including body weight loss, anorexia and decrease of physical activity, etc (Fig. 7.a). The body weight loss in the Exp+NaHCO_3_ group was also more obvious than in the Exp group.

**Figure 8:**
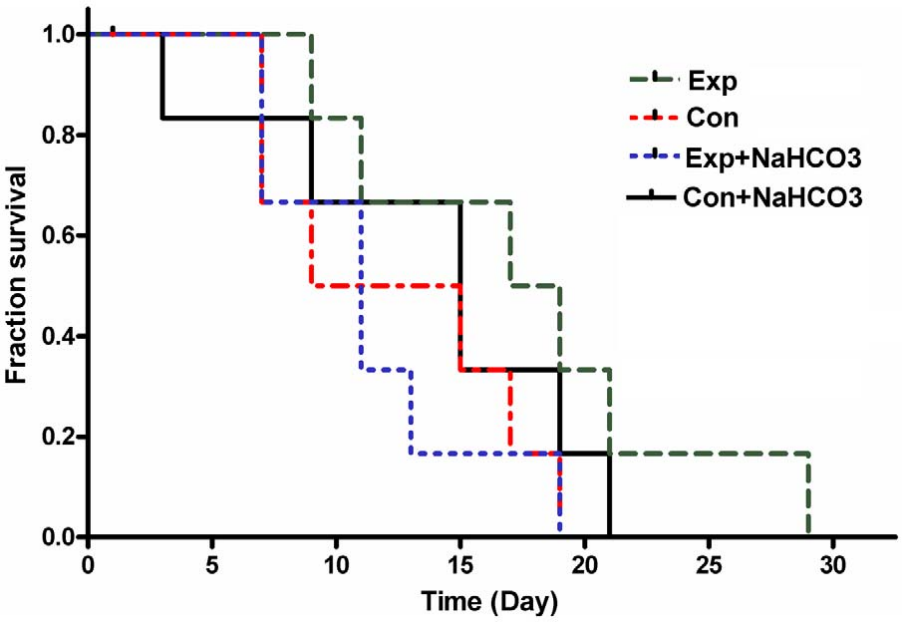
The survival curve of the mice in different groups. It can be observed that EXP+NaHCO_3_ failed to improve the survival time of the mice. Mice in the Exp group without NaHCO_3_ showed much longer survival time than those in the Exp+NaHCO_3_ group even though the tumor burden increases in these two groups were comparable.

The survival time in the Con and Con+NaHCO_3_ group was nearly the same. The Exp group exhibited longer survival time. However, the survival time in Exp+NaHCO_3_ group was not prolonged; it was even shorter than that in the Con+NaHCO_3_ group (Fig. 8.).

### The endocytosis of the NPs is the probable mechanism of the reversion of PIPDR

**Figure 9:**
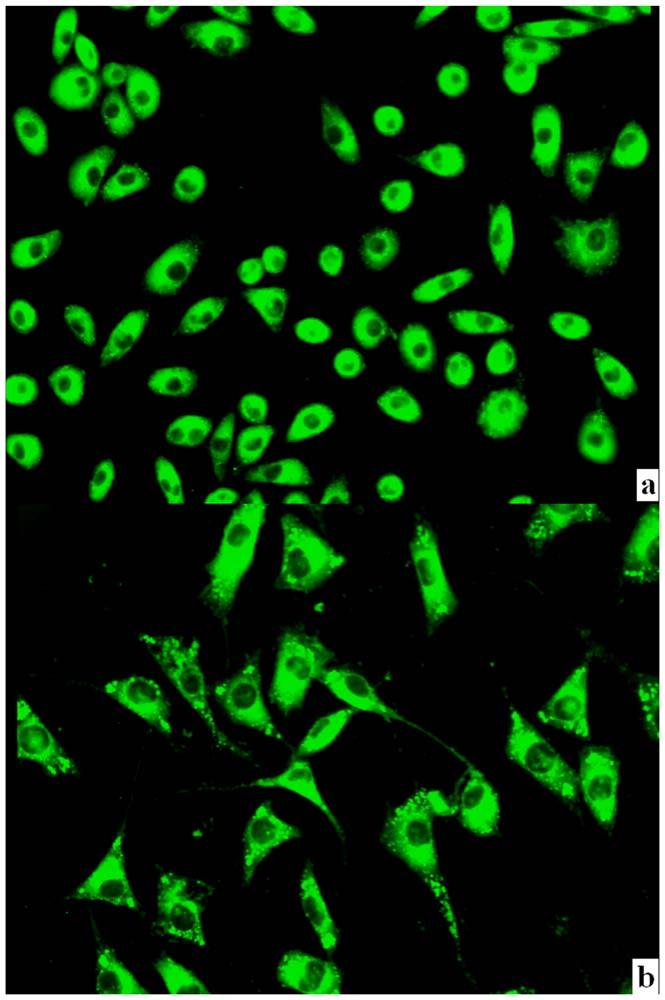
MKN-28 (**Fig. 9.a.**) and LoVo (**Fig. 9.b.**) cells incubated with coumarin-6-loaded NPs. It was obvious that the fluorescent signal deposited mostly in the peripheral region of cells, with little fluorescent signal observed inside the nuclei.

Cells (LoVo and MKN-28) co-incubated with coumarin-6-loaded NPs were observed using fluorescence microscopy and optical lens (200×). Fig. 9. shows that in both cell lines, coumarin-6 accumulated in the cytoplasm. In most of the cells, fluorescent dots could be found at the peripheral region of the cytoplasm (see the yellow arrows).

### The biocompatibility of PEG-PCL NPs

**Figure 10:**
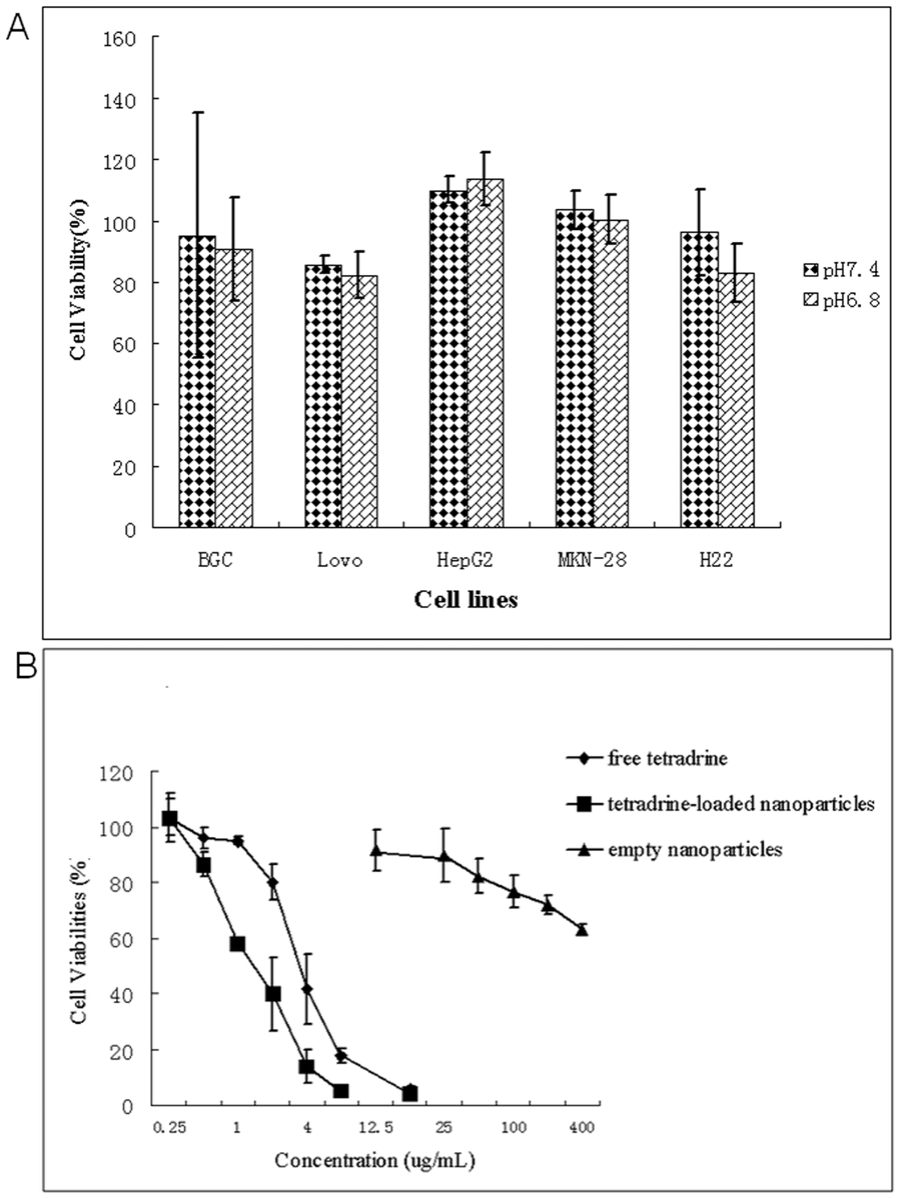
The in vitro toxicity of the blank NPs. Fig. 10.A. shows the toxicity of blank NPs on four tumor cell lines at a concentration of 400 µg/mL, and the data were presented as Mean±SD. Fig. 10.B. compared the inhibition rate of HUVEC treated with free Tet, Tet-NPs and blank NPs at different concentrations. The data were presented as Mean±SD. Where bars are not shown, SD is less than the height of the points.

The biocompatibility of the copolymer was evaluated in the above mentioned five tumor cell lines as well in HUVEC. Fig. 10.A. indicates that at a concentration of 400 µg/mL, the viabilities of the four cell lines were around 100%. Mild heterogeneity existed, however, with the viabilities of HepG_2_ and MKN-28 a little more than 100% and viabilities of BGC-823, LoVo and H_22_ sightly less than 100%. At different pH values, the viabilities of each cell line didn't showed significant difference (***P***>0.05). In HUVEC (Fig. 10.B.), at the same concentration, the blank nanoparticles exhibited far less toxicity compared with free Tet or Tet-loaded nanoparticles.

## Discussion

The rapid development of anticancer drug delivery systems has given birth to a variety of novel drug carriers characterized by copolymeric NPs [21]–[23]. Considerable research efforts have been given to the synthesis and evaluation of polymers as the drug carriers. The NPs without active targeting strategy are now the only category used clinically and commercially [23], [24]. However, these NPs usually exhibited prominent superiority only at in vivo studies. The in vitro effects of them were similar to or just slightly better than the free drug they load [25]–[27]. This discrepancy was merely attributed to the EPR effect and sustained release profile [4]–[7]. In this paper, when we focused on the tumor microenvironment, we figured out another important mechanism: the reversion of pH-induced physiological drug resistance (PIPDR).

Solid tumors are 3-dimentional structures composed of cancer cells and stromal cells as well as extracellular matrix and vascular network [8]. The physicochemical factors in tumor microenvironment are usually different from those in normal tissue. These altered physicochemical factors are important because they can influence the efficacy of the anticancer agents. Moreover, these factors (such as decreased pH, increased interstitial pressure, impaired penetration, etc.) exist widely in most of the tumors irrelevant to their primary site, pathological types or genetic backgrounds and can influence the effect of a great number of drugs regardless of their mechanisms. Therefore, tumor microenvironment has attracted more and more attentions in recent years [8], [28]. Among the microenvironmental factors, pH is of great importance as the decreased extracellular pH values are detected in most tumor tissues [12], as a result, focus on tumor extracellular pH may enhance the effectiveness of a great number of drugs in almost all kinds of tumors.

In this paper, we firstly confirmed PIPDR on different cancer cell lines using Tet. According to Fig. 4. and Table 2., the effectiveness of Tet was hindered significantly as a result of the decrease of tumor extracellular pH. Here we used Tet because it is not only a typical weakly basic drug but also an extract from Traditional Chinese Herbs, which indicates that PIPDR also hampers the effect of Traditional Chinese Medicine. Fig. 4. and Table 2. Also show that the PIPDR can be overcome by incorporating Tet into NPs. Besides MTT test, we also confirmed the effectiveness of NPs by apoptosis test and morphologic observation. These results also explain why the NPs without active targeting strategies only exhibit prominent superior effects at in vivo studies. The efficacy of free weakly basic drugs is similar to NPs at in vitro models with the extracellular pH of 7.4, however, at pH 6.8 (which resembles the actual situation in vivo), NPs exhibited significantly better effect.

It should also be noticed that the reversion of PIPDR works by increasing the intracellular drug concentration, so it could not improve the antitumor effect of drugs prominently by tens or hundreds folds. However, as pH is a physiochemical factor regardless of the genetic background of tumors or the type of antitumor agents, this new mechanism of the NPs has a wide influence on a large variety of anticancer agents as well as a large variety of solid tumors.

We further tested whether NPs can reverse PIPDR in vivo. Raghunand et al. have proved the existing of PIPDR in chemotherapeutics nearly 10 years ago and there have been a couple of studies on the reversion of PIPDR. However, most of those treatments failed to be brought into clinical use because of the present or potential toxicity or side effects [15], [17], [29]. The only in vivo study about the reversion of PIPDR was reported by Raghunand et al. [15] in which the bicarbonate-induced extracellular alkalinization leads to significant improvements in the therapeutic effectiveness of doxorubicin against MCF-7 xenografts. In the current study, we evaluated the effectiveness of Tet-NPs and compared their efficacy to Raghunand's methods. We evaluated the antitumor effect of free Tet and Tet-NPs under physiological pH of tumor and increased extracellular pH (by adding NaHCO_3_ into drinking water, according to Raghunand's report). The mice bore tumors at both of the axillary spaces, each receiving different agents, insuring that the extracellular pHs were identical within each subgroup. Therefore intratumoral injection was applied. Intratumoral injection can also reduce the influence of EPR effect [20], [27], [30] as the agents was delivered directed to the tumor site. In this case, other mechanisms apart from EPR effect would stand out.

At both pH values, Tet-NPs exhibited superior antitumor effect over free Tet. Moreover, the tumor volume increase in Tet-NP and Tet-NP+NaHCO_3_ subgroups were similar, which is compliant with in vitro studies, indicating that the antitumor effect of Tet-NPs was not influenced by tumor pH. ^18^FDG–PET-CT has been shown to be a reliable predictor of treatment response in clinical practice [27]. Here we applied PET-CT in the evaluation of early treatment response at the fifth day after administration of different agents. According to Fig. 6.a and Fig. 6.b, reduced tumor metabolism was found in all the 4 groups with Tet or Tet-NPs. When compared the subgroup free Tet to the subgroup free Tet+NaHCO_3_, the latter exhibited lower metabolism (P<0.05, Fig. 6.b), indicating that at day5, the antitumor effect in the subgroup free Tet+NaHCO_3_ was better and that of free Tet. Compared to the free Tet subgroups, Tet-NP subgroups exhibited lower metabolic activities at both extracellular tumor pH (P>0.05 between Tet-NP and Tet-NP+ NaHCO_3_ subgroups), which also confirmed the superiority of NPs regardless of pH values.

Both local and systemic side effects were found in this study including skin ulceration, body weight loss, anorexia and decrease of physical activity, etc (Fig. 7. and Fig.S5.), all of which were more prominent in the subgroups of free Tet. Actually, free Tet caused so severe skin ulceration that we have to choose a low dosage (with mild antitumor effect ) in this study. The alleviation of side effects of Tet-NPs also contributed to their high efficacy. Tet-NPs caused less side effects compared to free-Tet because Tet-NPs exhibits a sustainable release pattern, moreover, Tet-NPs enhanced the intracellular concentration of Tet and thus less Tet would accumulate in normal tissue. Therefore, compared to systemic side effects, the alleviation of local effect of Tet-NPs is much more prominent.

In free Tet subgroups (free Tet and Free Tet+NaHCO_3_), the retention of tumor growth was a little more prominent in the subgroups of free Tet+NaHCO_3_ on the tumor growth curve, which seemed different from Raghunand's report and our in vitro findings. This discrepancy attributes to three reasons: firstly, in this study, free Tet caused prominent side effects, which in turn impaired the effectiveness of Tet and secondly, the accuracy of the measurement of tumor diameters was hindered by the ulceration slightly but inevitably. PET-CT scanning indicated that the glucose metabolism of the tumors in free Tet+NaHCO_3_ subgroup was lower than that in the Tet free group, indicating that the efficacy of free Tet was superior in the subgroup with NaHCO_3_ at least during the first days. Thirdly, for free Tet, the side effect was positively correlated with its antitumor efficacy. The ulceration in the free Tet+NaHCO_3_ subgroup was more severe than the subgroup without NaHCO_3_ (Fig. 7.b. and Fig.S5.). All these indicate that for free Tet, the administration of NaHCO_3_ may exert effect on the improvement of the effectiveness of Tet, but this superiority may be hampered considerably by the side effects caused by free Tet.

As to Raghunand's report, the use of non-specific alkaloid may cause metabolic alkalinization and the increased extracellular pH may also sensitize the toxicity of the antitumor agents to normal cells such as intestinal epithelium [15]. Contrarily, the effect of NPs on the reversion of PIPDR is independent of local pH, and thus causes fewer side effects. Without the addition of another alkaloid, the NPs treatment will surely gain better patient compliance in future clinical use. In addition, these results together with the in vitro biocompatibility study (Fig. 10.) showed the safety of PEG-PCL nanopartciles.

The survival time is a comprehensive parameter evaluating the therapeutic effect. It can be observed from Fig. 8. that only the mice in the Exp group showed longer survival time even though the tumor volume increases in Exp and Exp+NaHCO_3_ groups were comparable. This finding also implicate that the mice may not actually benefit from NaHCO_3_ treatment.

For the ability of NPs to reverse PIPDR, there are two possible mechanisms: (1). Low extracellular pH accelerates the release of Tet from the NPs and (2). The NPs enter the cells via endocytosis rather than passive diffusion, which will avoid protonation of weakly basic drugs outside the cancer cell. Our study shows that the latter is probably the major mechanism.

Fig. 3. shows the in vitro release of Tet-loaded NPs at different pH values. At both pH values, the release profile of the NPs was similar: An initial burst of more than 50% (pH 6.8) and 30% (pH 7.4) release in the first 4 hrs. Then the release rate of Tet slowed down and presented a sustained manner. During the tested time span, the release of Tet from the NPs at pH 6.8 was rapid than that of pH 7.4. However, the gap between the release proportions was narrowed gradually with time. At 120 h, more than 90% of the Tet was released at both pH values (90% for pH 7.4 and 92% for pH 6.8). Generally, the release profile was more sustainable at pH 7.4. This difference in release rate indicates that Tet in the NPs will released in a targeted pattern at the tumor site where the regional pH is lower. However, as the difference was not prominent, with the maxium difference about 18%, this may not be the main mechanism of the NP's reversion of PIPDR. This can also be concluded from the in vitro toxicity study: for two cell lines (BGC-823 and HepG_2_), the antitumor effect of the NPs didn't increase at pH 6.8. On the other hand, the NPs reversed the decrease of antitumor effect of free Tet at lower pH with significant differences (see Table 2.).

We also looked into the other possible mechanisms by particle cellular uptake studies. From Fig. 9.a and Fig. 9.b., we found that after incubation with cancer cells for 4 hrs, fluorescent signals of Coumarin-6 could be observed inside the cells. As Coumarin-6 was insoluble in water, the intracellular Coumarin-6 was that originally incorporated in the NPs, which indicated that drugs could enter the cancer cell using NPs as the carrier. This is in accordance with the findings that NPs could enter the cancer cell via endocytosis [16], [18], [19]. In this case, the Tet loaded in the NPs would be delivered across the cell membrane in its unionized form. As the pH value inside the cell was normal (around 7.4), free Tet in the cytoplasm would remain this active form and kill the cancer cell efficiently.

There are three two points we want to indicate about this study. Firstly, an important branch of targeted drug delivery strategy is pH targeting. For most pH-sensitive drug carriers delivering anticancer agents, low pH causes acceleration of drug release usually extracellularly [31]–[32]. If the loaded drugs are weakly basic, the released drugs may protonated and accumulate outside the cells. So we hypothesize here that pH-targeting may not be so efficacy as it has been supposed. Nevertheless, it still works because deformation of drug carriers triggered by pH leads to accumulation of NPs and drugs in the tumor tissue, which certainly increase the specificity of drug delivery and could be more effective than free drugs. Secondly, as PIPDR is in close relation to the Pka of the drugs, the choice of appropriate drugs for certain carriers should be paid close attention to. For example, pH sensitive drug carriers are more suitable for the delivery of drugs with Pka less than 7 because the effectiveness of weakly acidic drugs is enhanced at extracellular pH of tumors while carriers of no pH targeting traits are advised to be chosen for delivery of weakly basic drugs. Also, the efficacy of new drug delivery system is recommended to be evaluated at different extracellular pH values in the in vitro study.

### Conclusion

In this study, we raised the definition of pH-induced physiological drug resistance (PIPDR) and confirmed this phenomenon by experiment. We further proved that copolymeric nanoparticles (NPs) could reverse PIPDR in vitro and in vivo by a series of studies. To our best knowledge, this is among the first reports concentrating on the relationship of NPs and the physiochemical factors at tumor tissue, which raised some new considerations on the choice of drugs for different drug carriers as well as pH-responsive drug delivery systems. We believe that the findings in this report may exert wide influence because this new mechanism affects a large number of antitumor agents and copolymeric drug carriers, though more studies are still needed to further confirm this mechanism on other drugs and/or other polymeric drug carriers.

## Supporting Information

Methods S1 Synthesis of mPEG and PCL block copolymers, characterization of mPEG-PCL copolymers, preparation of mPEG-PCL nanoparticles, and characterization of nanoparticles (including Particle size and morphology evaluation and drug loading content and encapsulation efficiency).
(DOC)

Results S1 Copolymer synthesis and characterization, size distribution, morphology studies, and drug loading content and encapsulation efficiency.
(DOC)

Figure S1 **^1^H-NMR spectra of methoxy poly(ethylene glycol)–polycaprolactone (mPEG–PCL).**
(TIF)

Figure S2 **Gel permeation chromatography (GPC) of mPEG-PCL.**
(TIF)

Figure S3 **TEM micrograph (Fig.S3.A.) and AFM micrograph (Fig.S3.B.) of the NPs.**
(TIF)

Figure S4 **Tumor volume of established H22 xenografts in ICR mice during therapy under different treatments (All the 8 subgroups).** Mice were treated with different protocols (Table 1.). Different agents were delivered through intratumoral pathway. Data are presented as mean ± SD.
(TIF)

Figure S5 **The ulceration caused by Tet.** The ulceration at the tumor was evaluated every other day according to Table.S4. It can be found that free Tet at the same dose caused prominent ulceration. The ulceration in the subgroup Tet free+NaHCO3 was more severe than that in the Tet free subgroup. However, for the two subgroups receiving Tet-NPs, no ulceration was found except the first day after treatment, indicating that Tet-NPs are able to attenuate the side effects of Tet prominently.
(TIF)

Table S1 Molecular weights measured by GPC and 1H-NMR.
(DOC)

Table S2 Diameters of the NPs determined by DLS.
(DOC)

Table S3 The influence of drug feeding on drug loading content and encapsulation efficiency.
(DOC)

Table S4 Scoring for local ulceration.
(DOC)

## References

[1] Ibrahim NK, Samuels B, Page R, Doval D, Patel KM (2005). Multicenter phase II trial of ABI-007, an albumin-bound paclitaxel, in women with metastatic breast cancer.. J Clin Oncol.

[2] Lee MJ, Veiseh O, Bhattarai N, Sun C, Hansen SJ, Ditzler S (2010). Rapid pharmacokinetic and biodistribution studies using cholorotoxin-conjugated iron oxide nanoparticles: a novel non-radioactive method.. PLoS One.

[3] Nishiyama N (2007). Nanomedicine: nanocarriers shape up for long life.. Nat Nanotechnol.

[4] Sanhai WR, Sakamoto JH, Canady R, Ferrari M (2008). Seven challenges for nanomedicine.. Nat Nanotechnol.

[5] Hall JB, Dobrovolskaia MA, Patri AK, McNeil SE (2007). Characterization of nanoparticles for therapeutics.. Nanomedicine.

[6] Hoffman AS (2008). The origins and evolution of “controlled” drug delivery systems.. J Control Release.

[7] Matsumura Y (2008). Poly (amino acid) micelle nanocarriers in preclinical and clinical studies.. Adv Drug Deliv Rev.

[8] Tredan O, Galmarini CM, Patel K, Tannock IF (2007). Drug resistance and the solid tumor microenvironment.. J Natl Cancer Inst.

[9] Raghunand N, Altbach MI, van Sluis R, Baggett B, Taylor CW (1999). Plasmalemmal pH-gradients in drug-sensitive and drug-resistant MCF-7 human breast carcinoma xenografts measured by ^31^P magnetic resonance spectroscopy.. Biochem Pharmacol.

[10] Raghunand N, Gillies RJ (2000). pH and drug resistance in tumors.. Drug Resist Updat.

[11] Olive KP, Jacobetz MA, Davidson CJ, Gopinathan A, McIntyre D (2009). Inhibition of Hedgehog signaling enhances delivery of chemotherapy in a mouse model of pancreatic cancer.. Science.

[12] Gerweck LE, Seetharaman K (1996). Cellular pH gradient in tumor versus normal tissue: potential exploitation for the treatment of cancer.. Cancer Res.

[13] Vukovic V, Tannock IF (1997). Influence of low pH on cytotoxicity of paclitaxel, mitoxantrone and topotecan.. Br J Cancer.

[14] Raghunand N, Gillies RJ (2001). pH and chemotherapy.. Novartis Found Symp.

[15] Raghunand N, He X, van SR, Mahoney B, Baggett B (1999). Enhancement of chemotherapy by manipulation of tumour pH.. Br J Cancer.

[16] Wang Y, Bansal V, Zelikin AN, Caruso F (2008). Templated synthesis of single-component polymer capsules and their application in drug delivery.. Nano Lett.

[17] Lee CM, Tannock IF (2006). Inhibition of endosomal sequestration of basic anticancer drugs: influence on cytotoxicity and tissue penetration.. Br J Cancer.

[18] Hillaireau H, Couvreur P (2009). Nanocarriers' entry into the cell: relevance to drug delivery.. Cell Mol Life Sci.

[19] Rosen H, Abribat T (2005). The rise and rise of drug delivery.. Nat Rev Drug Discov.

[20] Li R, Li X, Xie L, Ding D, Hu Y (2009). Preparation and evaluation of PEG-PCL nanoparticles for local tetradrine delivery.. Int J Pharm.

[21] Segal E, Pan H, Ofek P, Udagawa T, Kopecková P (2009). Targeting angiogenesis-dependent calcified neoplasms using combined polymer therapeutics.. PLoS One.

[22] McCarron PA, Faheem AM (2010). Nanomedicine-based cancer targeting: a new weapon in an old war.. Nanomedicine.

[23] Nyman DW, Campbell KJ, Hersh E, Long K, Richardson K (2005). Phase I and pharmacokinetics trial of ABI-007, a novel nanoparticle formulation of paclitaxel in patients with advanced nonhematologic malignancies.. J Clin Oncol.

[24] Bourgeois H, Ferru A, Lortholary A, Delozier T, Boisdron-Celle M (2006). Phase I-II study of pegylated liposomal doxorubicin combined with weekly paclitaxel as first-line treatment in patients with metastatic breast cancer.. Am J Clin Oncol.

[25] Zhu Z, Li Y, Li X, Li R, Jia Z, Liu B (2010). Paclitaxel-loaded poly(N-vinylpyrrolidone)-b-poly(epsilon-caprolactone) nanoparticles: preparation and antitumor activity in vivo.. J Control Release.

[26] Xiao K, Luo J, Fowler WL, Li Y, Lee JS (2009). A self-assembling nanoparticle for paclitaxel delivery in ovarian cancer.. Biomaterials.

[27] Li X, Li R, Qian X, Ding Y, Tu Y (2008). Superior antitumor efficiency of cisplatin-loaded nanoparticles by intratumoral delivery with decreased tumor metabolism rate.. Eur J Pharm Biopharm.

[28] Minchinton AI, Tannock IF (2006). Drug penetration in solid tumours.. Nat Rev Cancer.

[29] Rotin D, Wan P, Grinstein S, Tannock I (1987). Cytotoxicity of compounds that interfere with the regulation of intracellular pH: a potential new class of anticancer drugs.. Cancer Res.

[30] Maeda H, Wu J, Sawa T, Matsumura Y, Hori K (2000). Tumor vascular permeability and the EPR effect in macromolecular therapeutics: a review.. J Control Release.

[31] Kojima C (2010). Design of stimuli-responsive dendrimers.. Expert Opin Drug Deliv.

[32] Liu R, Li D, He B, Xu X, Sheng M (2011). Anti-tumor drug delivery of pH-sensitive poly(ethylene glycol)-poly(L-histidine-)-poly(L-lactide) nanoparticles.. J Control Release.

