# Supplementary material for: Reversion of pH-Induced Physiological Drug Resistance: A Novel Function of Copolymeric Nanoparticles
Source: PLoS One. 2011 Sep 26;6(9):e24172. doi: 10.1371/journal.pone.0024172 (PMC3180282; doi:10.1371/journal.pone.0024172)
Supplement: Methods S1 — Synthesis of mPEG and PCL block copolymers, characterization of mPEG-PCL copolymers, preparation of mPEG-PCL nanoparticles, and characterization of nanoparticles (including Particle size and morphology evaluation and drug loading content and encapsulation efficiency). (DOC) [file pone.0024172.s001.doc]

**Methods**

***Synthesis of mPEG and PCL block copolymers***

The mPEG and PCL block copolymer was synthesized by a ring opening copolymerization mainly as we previously described with slight modification[1, 2] . Briefly, predetermined amount of ε-CL was added into a polymerization tube containing mPEG and stannous octoate (0.1% wt/wt). The tube was then connected to a vacuum system, sealed off, and placed in an oil bath at 130 °C for 48 h. At the end of the polymerization, the crude copolymers were dissolved with dichloromethane (DCM) and precipitated into an excess amount of cold methanol to remove the un-reacted monomer and oligomer. The precipitates were then filtered and washed with water several times before thoroughly dried at reduced pressure.

***Characterization of mPEG-PCL copolymers***

Examination of the mPEG-PCL copolymers was conducted on a Bruker AM-300 spectrometer using CDCl_3_ as the solvent. The composition and molecular weight (M_n_) of each copolymer were calculated from the integration value of the peaks in the ^1^HNMR spectra that correspond to the protons on CL and PEG..

Molecular weight of the copolymers was also determined using a Waters GPC 244 system with tetrahydrofuran (THF) as the eluent at a flow rate of 1 mL/min. Calibration was accomplished using monodispersed polystyrene standards with a molecular weight range from 800 to 124,000 g/mol.

***Preparation of mPEG-PCL nanoparticles***

Tet-loaded nanoparticles were prepared by single O/W emulsion and solvent evaporation method according to the description of Tewes et al. [3] with modification. Brieiefly, 20mg of mPEG-PCL and a certain amount of tetradrine were dissolved in 1mL of methylene chloride. The mixture was emulsified in 3 ml of aqueous poly(vinyl alcohol) (PVA) solution at 5% (w/v) by sonication (XL2000，Misonix，USA) for 30 s (17.5 W) to obtain an O/W emulsion. This emulsion was then diluted in 8 ml of aqueous solution containing 1% (w/v) PVA and left under mechanical stirring for 2 hours to remove methylene chloride. The resulted solution was filtered to remove non-incorporated tetradrine. Blank nanoparticles were produced in a similar manner without adding drug. Finally, nanoparticle suspensions were freeze dried and stored at 4°C.

***Characterization of nanoparticles***

***Particle size and morphology evaluation***

Mean diameter and size distribution were measured by photon correlation spectroscopy (DLS) using a Brookheaven BI-9000AT instrument (Brookheaven Instruments Corporation, NY, USA). All measurements were performed at 25°C. Calculation of the size and polydispersity indices was achieved by the software provided by the manufacturer. The diameters mean values were calculated from the measurements performed at least in triplicate.

Morphological examination of the nanoparticles was conducted using JEM-100S (Japan) transmission electron microscope (TEM). One drop of nanoparticle suspension was placed on a copper grid covered with nitrocellulose membrane and air-dried before negative staining with phosphotungstic sodium solution (1% w/v). Atomic force microscope (AFM) (SPI3800, Seiko Instruments, Japan) was used to study the surface morphology of nanoparticles in a greater detail. One drop of properly diluted nanoparticle suspension was placed on the surface of a clean silicon wafer and dried under nitrogen flow at room temperature. The AFM observation was performed with a 20 μm scanner in tapping mode.

***Drug loading content and encapsulation efficiency***

To determine the drug loading content, the freeze-dried powder of the nanoparticles was dissolved in acetonitrile. The tetradrine concentration in the resulted acetonitrile solution was then determined by the ultraviolet absorption at the wavelength of 280.4nm, a strong absorption band of tetradrine with reference to a calibration curve on a Shimadzu UV3100 spectrophotometer (Shimadzu, Japan). Then the total amount of the drug contained in the nanoparticle could be calculated.

Drug loading content and encapsulation efficiency were obtained by the following equations:

$\begin{matrix} \text{Drug loading content (\%)}= \\ \frac{\text{Weight of the drug in nanoparticles}}{\text{Weight of the drug in nanoparticles}+\text{Weight of the copolymer}}\times\text{100\%} \end{matrix}$ (1)

$\text{Encapsulation efficiency (\%)}=\frac{\text{Weight of the drug in nanoparticles}}{\text{ Weight of the feeding drugs}}\times\text{100\%}$ (2)

**References**

1. Zhang L, Hu Y, Jiang X, Yang C, Lu W et al (2004) Camptothecin derivative-loaded poly(caprolactone-co-lactide)-b-PEG-b-poly(caprolactone-co-lactide) nanoparticles and their biodistribution in mice. J Control Release 96: 135-148.

2. Hu Y, Jiang X, Ding Y, Zhang L, Yang C et al (2003) Preparation and drug release behaviors of nimodipine-loaded poly(caprolactone)-poly(ethylene oxide)-polylactide amphiphilic copolymer nanoparticles. Biomaterials 24: 2395-2404.

3. Tewes F, Munnier E, Antoon B, Ngaboni Okassa L, Cohen-Jonathan S et al. (2007) Comparative study of doxorubicin-loaded poly(lactide-co-glycolide) nanoparticles prepared by single and double emulsion methods. Eur J Pharm Biopharm 66: 488-492.
