# Supplementary material for: Reversion of pH-Induced Physiological Drug Resistance: A Novel Function of Copolymeric Nanoparticles
Source: PLoS One. 2011 Sep 26;6(9):e24172. doi: 10.1371/journal.pone.0024172 (PMC3180282; doi:10.1371/journal.pone.0024172)
Supplement: Results S1 — Copolymer synthesis and characterization, size distribution, morphology studies, and drug loading content and encapsulation efficiency. (DOC) [file pone.0024172.s002.doc]

**Results**

***Copolymer synthesis and characterization***

The molecular weight of the polymers can be obtained from ^1^H-NMR data by comparing the peak intensities of the methylene protons of the oxyethylene units of PEG to the methylene of PCL (Suppl Fig.1.). The feed ratio and calculated molecular weight were summarized in Table. 1. The nomenclature of the two copolymers was referred to the feed ratio.

In GPC analysis, only one peak appeared in the GPC curve, which indicated that all the impurities were removed after purification. (Suppl Fig.2.) The molecular weight and molecular weight distribution of the two samples are also listed in Suppl Tab. 1. The polydispersity of the copolymer (defined as the ratio of weight-average molecular weight to the number-average molecular weight) was 1.69. The number-average molecular weight obtained from the GPC chromatogram confirmed the calculated NMR values.

***Size distribution***

We compared the particle size and size distribution of empty nanoparticles and Tet-loaded nanoparticles before and after freeze-dry. The results are listed in Suppl Table.2.

There was no significant difference between empty nanoparticles and Tet-loaded nanoparticles as to the mean diameter or polydispersity (***P***>0.05). Slight difference of diameters for the Tet-loaded nanoparticles(***P***<0.05) was observed before and after freeze-dry.

***Morphology studies***

Suppl Fig. 3.A shows the TEM photograph of mPEG-PCL nanoparticles. It could be observed that most of the copolymer nanoparticles had a regular spherical shape and the size was around 300nm. By close observation of TEM photographs, it was noticed that bright and dark regions could be seen in these nanoparticles.

On the AFM photograph (Suppl Fig.3.B), The size and the spherical shape of the nanoparticles correlated well with the TEM results.

***Drug loading content and encapsulation efficiency***

Suppl Table.3. shows drug loading content and encapsulation efficiency as a function of feeding drug/copolymer ratio. The encapsulation efficiency decreased with the increase of feeding drug/copolymer ratio. When the ratio increases from 0.25 to 0.375, drug loading content increases. The drug loading content decreases as the drug/copolymer ratio increases from 0.375 to 0.5. The drug/copolymer ratio of 0.375 was chosen in the following study.
