# Supplementary figures and images for: Reversion of pH-Induced Physiological Drug Resistance: A Novel Function of Copolymeric Nanoparticles
Source: PLoS One. 2011 Sep 26;6(9):e24172. doi: 10.1371/journal.pone.0024172 (PMC3180282; doi:10.1371/journal.pone.0024172)

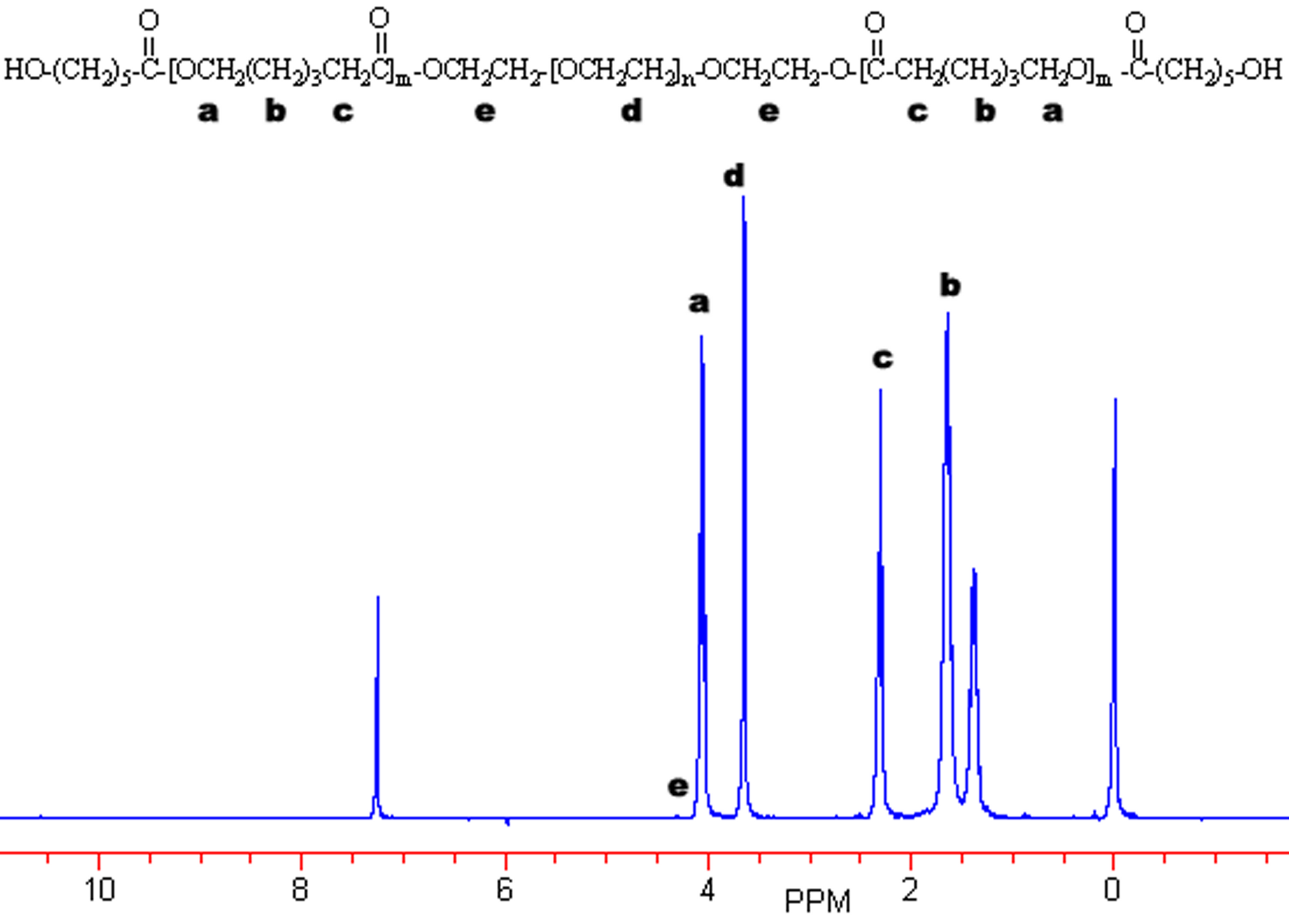

Supplement: Figure S1 — 1H-NMR spectra of methoxy poly(ethylene glycol)–polycaprolactone (mPEG–PCL). (TIF) [file pone.0024172.s003.tif]

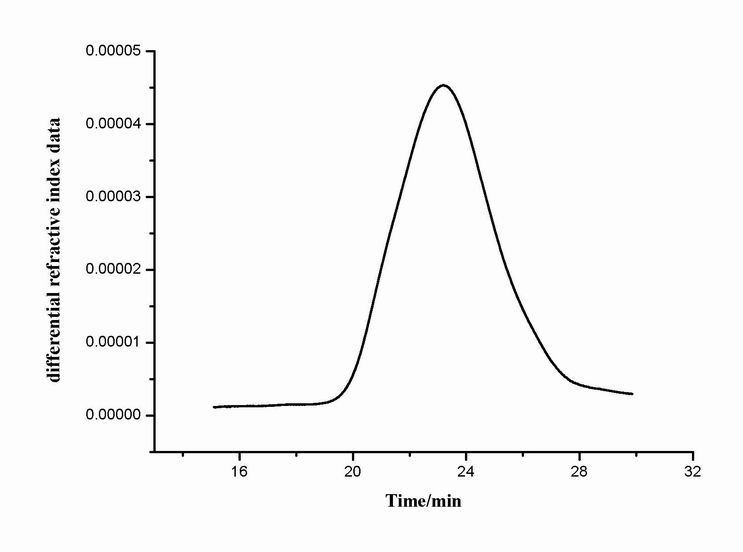

Supplement: Figure S2 — Gel permeation chromatography (GPC) of mPEG-PCL. (TIF) [file pone.0024172.s004.tif]

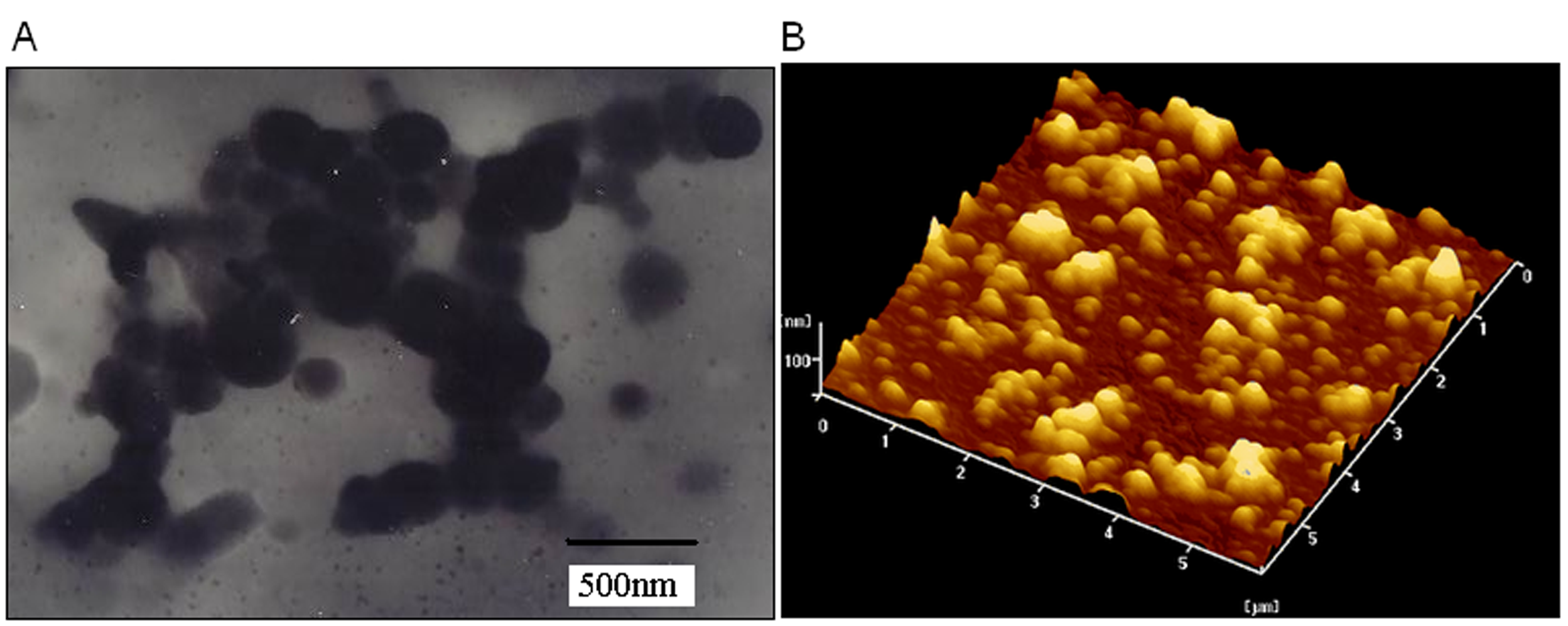

Supplement: Figure S3 — TEM micrograph (Fig.S3.A.) and AFM micrograph (Fig.S3.B.) of the NPs. (TIF) [file pone.0024172.s005.tif]

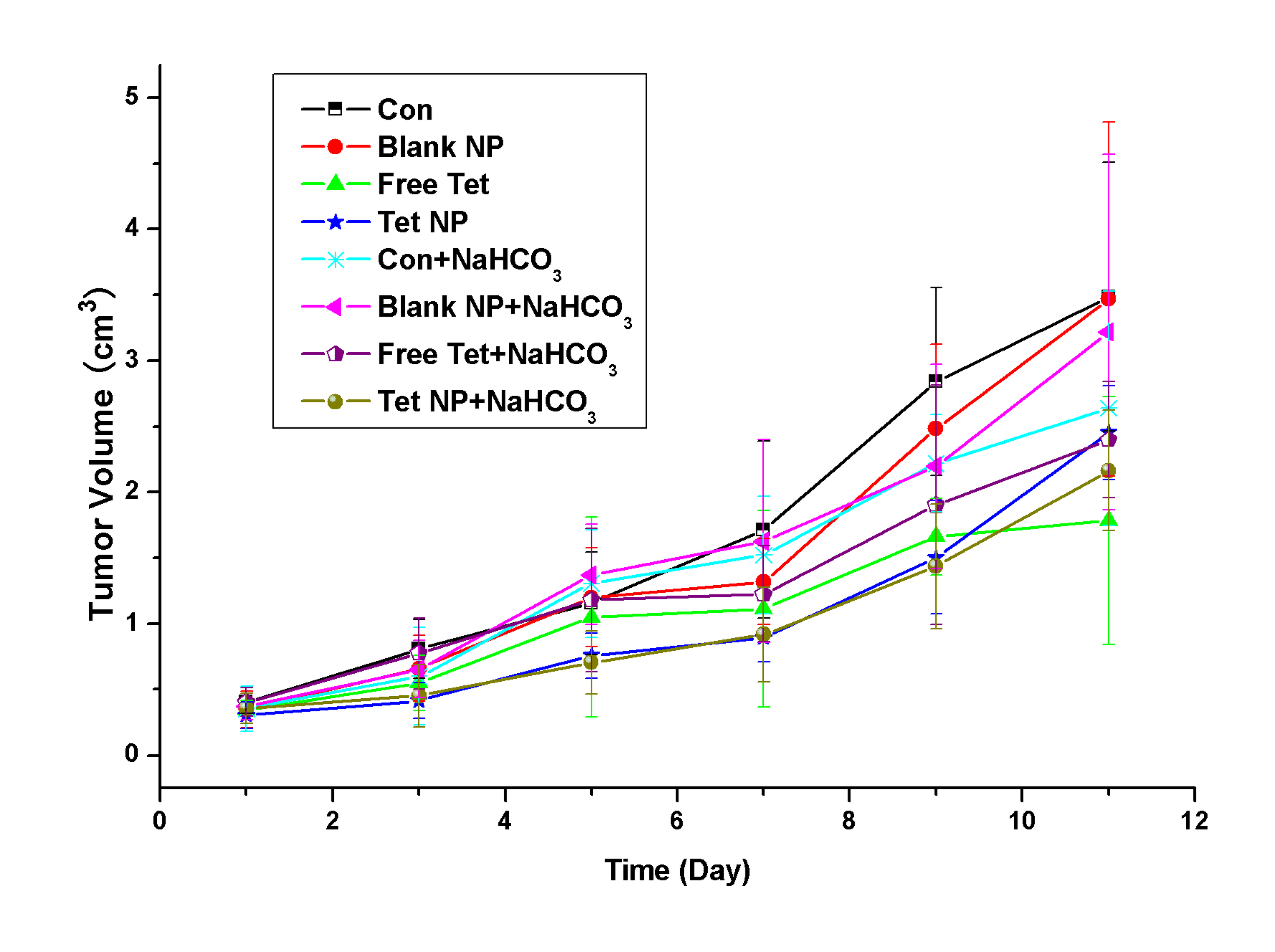

Supplement: Figure S4 — Tumor volume of established H22 xenografts in ICR mice during therapy under different treatments (All the 8 subgroups). Mice were treated with different protocols (Table 1.). Different agents were delivered through intratumoral pathway. Data are presented as mean ± SD. (TIF) [file pone.0024172.s006.tif]

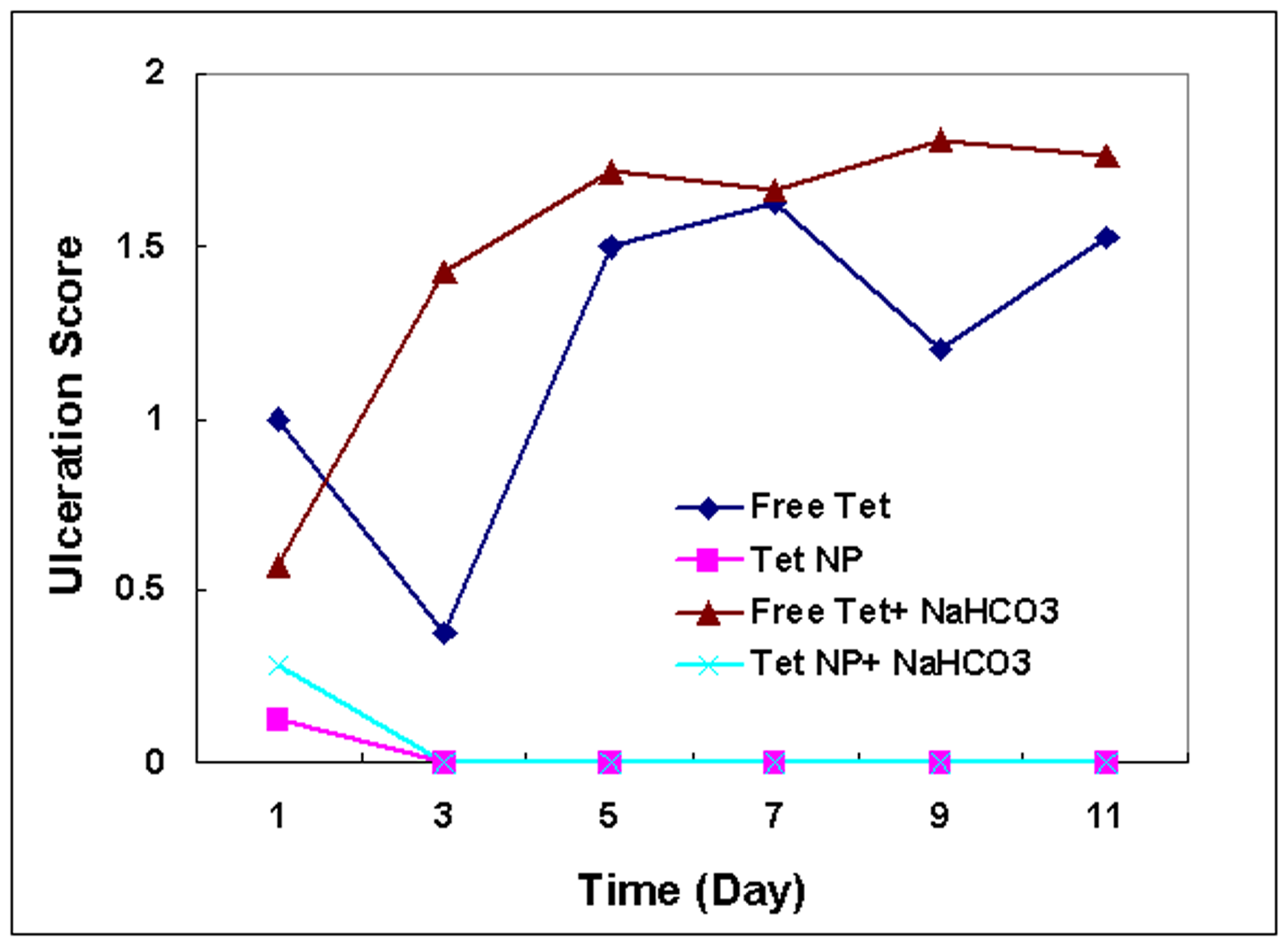

Supplement: Figure S5 — The ulceration caused by Tet. The ulceration at the tumor was evaluated every other day according to Table.S4. It can be found that free Tet at the same dose caused prominent ulceration. The ulceration in the subgroup Tet free+NaHCO3 was more severe than that in the Tet free subgroup. However, for the two subgroups receiving Tet-NPs, no ulceration was found except the first day after treatment, indicating that Tet-NPs are able to attenuate the side effects of Tet prominently. (TIF) [file pone.0024172.s007.tif]
