# Supplementary material for: Reversion of pH-Induced Physiological Drug Resistance: A Novel Function of Copolymeric Nanoparticles
Source: PLoS One. 2011 Sep 26;6(9):e24172. doi: 10.1371/journal.pone.0024172 (PMC3180282; doi:10.1371/journal.pone.0024172)
Supplement: Table S1 — Molecular weights measured by GPC and 1H-NMR. (DOC) [file pone.0024172.s008.doc]

Table.S1.

**Molecular weights measured by GPC and ^1^H-NMR**

|  | (nCL/nEG)^a^ | (nCL/nEG)^b^ | Mn^b^(g/mol) | Mw^c^(g/mol) | Mn^c^(g/mol) | d(Mw^c^ /Mn^c^) |
| --- | --- | --- | --- | --- | --- | --- |
| mPEG-PCL | 1.93 | 1.90 | 23691 | 43763 | 25826 | 1.69 |

**^a^** Feeding

**b** Determined by ^1^H-NMR.

**^c^** Determined by GPC.
