# Supplementary material for: Reversion of pH-Induced Physiological Drug Resistance: A Novel Function of Copolymeric Nanoparticles
Source: PLoS One. 2011 Sep 26;6(9):e24172. doi: 10.1371/journal.pone.0024172 (PMC3180282; doi:10.1371/journal.pone.0024172)
Supplement: Table S2 — Diameters of the NPs determined by DLS. (DOC) [file pone.0024172.s009.doc]

Table.S2.

Diameters of the NPs determined by DLS

| nanoparticles | | diameter（nm） | polydispersity |
| --- | --- | --- | --- |
| Tet-NPs | Before freeze-dry | 272.6±3.2 | 0.112±0.091 |
|  | After freeze-dry | 285.8±4.2* | 0.054±0.085 |
| Blank NPs | Before freeze-dry | 281.5±3.8 | 0.204±0.021 |
|  | After freeze-dry | 287.6±6.0 | 0.160±0.071 |

* paired T-test: before VS after freeze-dry, Tet-loaded nanoparticles *P*＝0.012.
