# Supplementary material for: Reversion of pH-Induced Physiological Drug Resistance: A Novel Function of Copolymeric Nanoparticles
Source: PLoS One. 2011 Sep 26;6(9):e24172. doi: 10.1371/journal.pone.0024172 (PMC3180282; doi:10.1371/journal.pone.0024172)
Supplement: Table S3 — The influence of drug feeding on drug loading content and encapsulation efficiency. (DOC) [file pone.0024172.s010.doc]

Table.S3.

The influence of drug feeding on drug loading content and encapsulation efficiency

| Feeding Drugs (mg) | 5mg | 7.5mg | 10mg |
| --- | --- | --- | --- |
| Feeding drug/copolymer ratio | 0.25 | 0.375 | 0.5 |
| Drug loading content | 12.02% | 14.38% | 13.01% |
| Encapsulation efficiency | 54.64% | 44.93% | 29.88% |
