# Supplementary material for: Reversion of pH-Induced Physiological Drug Resistance: A Novel Function of Copolymeric Nanoparticles
Source: PLoS One. 2011 Sep 26;6(9):e24172. doi: 10.1371/journal.pone.0024172 (PMC3180282; doi:10.1371/journal.pone.0024172)
Supplement: Table S4 — Scoring for local ulceration. (DOC) [file pone.0024172.s011.doc]

Table.S4.

Scoring for local ulceration

| Score | ulceration |
| --- | --- |
| 0 | No ulceration |
| 1 | The area of ulceration≤1/3 surface area of the tumor |
| 2 | The area of ulceration occupies 1/3-1/2 surface area of the tumor |
| 3 | The area of ulceration≥1/2 surface area of the tumor |

We scored the ulceration in order to analyze the side effect of Tet. The severity of the ulceration was mainly evaluated according to its area.
